# Effectiveness of strategies to facilitate uptake or implementation of complex interventions: A systematic review of reviews

**DOI:** 10.1186/1748-5908-10-S1-A67

**Published:** 2015-08-20

**Authors:** Rosa Lau, Fiona Stevenson, Bie Nio Ong, Krysia Dziedzic, Shaun Treweek, Sandra Eldridge, Hazel Everitt, Anne Kennedy, Nadeem Qureshi, Anne Rogers, Richard Peacock, Elizabeth Murray

**Affiliations:** 1 E-Health Unit, Department of Primary Care and Population Health, University College London, London, NW3 2PF, UK; 2 Arthritis Research UK Primary Care Centre, Research Institute for Primary Care Sciences and Health Sciences, Keele University, Keele, Staffordshire, ST5 5BG, UK; 3 Health Services Research Unit, University of Aberdeen, Aberdeen, AB25 2ZD, UK; 4 Centre for Primary Care and Public Health, Queen Mary University of London, London, E1 2AT, UK; 5 Primary Care and Population Sciences, Faculty of Medicine, University of Southampton, Southampton, SO16 5ST, UK; 6 Faculty of Health Sciences, NIHR CLAHRC Wessex, University of Southampton, Southampton, SO17 1BJ, UK; 7 Division of Primary Care, University of Nottingham, Nottingham, NG7 2RD, UK; 8 Archway Healthcare Library, London, N19 5NF, UK

## Introduction

Research has consistently shown that many effective complex interventions are not taken up in practice. Getting evidence or complex interventions implemented into routine practice is often a challenge, particularly in primary care. Complex interventions are defined as interventions with several interacting components, e.g. prescribing decision support to aid guideline implementation, web-based self-management programme for people with type 2 diabetes. To bridge this evidence-to-practice gap, it is important to use effective methods/strategies to optimize implementation.

## Aim/objectives

Assess the effectiveness of different strategies (single or multifaceted) for optimizing implementation of complex interventions; Assess the effects of strategies in different clinical areas (e.g. prevention, guideline, prescribing); Identify active components that contribute towards effective implementation; Describe cost-effectiveness evidence of these strategies.

## Method

Five electronic databases were searched until December 2013. Citations and full-text papers were independently screened by two reviewers against pre-defined selection criteria [population: primary care in developed countries; intervention: implementation of complex interventions, by using single/multifaceted implementation strategies; comparison(s): usual care, no strategy, another strategy (single/multifaceted); outcomes: degree of implementation, e.g. process, professionals' behaviour or performance; study design: reviews]. Data were extracted using standardized data abstraction forms. A multi-step systematic process was developed; results were described narratively and the synthesis was guided by the pre-defined research questions.

## Findings

91 reviews were included. For dichotomous outcomes, effects of educational outreach visits, audit & feedback, educational meetings and computerized reminders were small-moderate (some more variable than others). Multifaceted strategies were not necessarily better than single strategies. However, multifaceted strategies including organizational interventions (redefined role, enhanced multidisciplinary team work) appeared to be more effective in changing practice. Active (and inactive) components that contributed towards the effectiveness of implementation were identified. There was limited evidence on the cost-effectiveness of using these implementation strategies.

How the research advances dissemination and implementation research

This work will provide a comprehensive overview of the topic by providing a deeper understanding of how to implement evidence-based approaches to improve service delivery and quality of patient care; and inspire individuals to think differently when planning and implementing a complex intervention in primary care. Implications for practice and future research were drawn from the findings of this review.

## Funding

This Project (SPCR FR4 project number: 122) is funded by the National Institute of Health Research (NIHR) School for Primary Care Research (SPCR). This paper presents independent research funded by the National Institute of Health Research (NIHR). The views expressed are those of the author(s) and not necessarily those of the NHS, the NIHR or the Department of Health.

